# Evidence for a fading affect bias in subjectively assessed affect changes in autobiographical memory

**DOI:** 10.3389/fpsyg.2025.1608751

**Published:** 2025-07-28

**Authors:** Sophie Hoehne, Daniel Zimprich

**Affiliations:** Department of Developmental Psychology, Institute of Psychology and Education, Ulm University, Ulm, Germany

**Keywords:** autobiographical memory, fading affect bias, subjective change, emotionality, FAB

## Abstract

**Introduction:**

Over time, autobiographical memories (AMs) can decrease in affect intensity (fading affect), increase in affect intensity (flourishing affect), remain the same (fixed affect), or change valence (flexible affect). In this regard, the fading affect bias (FAB) names the phenomenon that, on average, negative AMs’ affect intensity fades more strongly and more rapidly over time than positive AMs’ affect intensity, which sometimes even increases on average. The FAB has most often been investigated using difference scores between retrospective ratings of AMs’ initial affect intensity and their affect intensity from a current perspective (calculated change). The goal of the present study was to complement this previous research by additionally investigating the FAB in *subjective* change, that is, the directly assessed, subjectively perceived direction of affect change in an AM over time.

**Method:**

Mixed-effects multinominal models were used to predict the probabilities of the different types of subjective change, which were then compared with their likelihood in calculated change. Moreover, the influence of initial intensity, neuroticism, and life satisfaction was compared between subjective change and calculated change. Analyses were based on 12,314 AMs reported by 2,163 adults aged 18–98 years.

**Results:**

A clear FAB was found for both subjective and calculated change. Neuroticism and life satisfaction showed comparable effects on subjective and calculated change, but the effects of initial intensity differed. Specifically, the effects of initial intensity on the probability of flourishing and flexible affect in subjective change were in the same direction as for calculated change, albeit with substantially weaker effect sizes. Moreover, only for subjective change did initial intensity have an effect on the likelihood of fading affect (negative effect).

**Discussion:**

The present findings extend previous knowledge of the FAB and its predictors, particularly with regard to initial intensity. Moreover, the present study supports the idea of an emotion-regulating, self-serving functionality of autobiographical memory, also within subjective emotional changes, and sheds an optimistic light on the appropriateness of retrospective designs in FAB studies.

## 1 Introduction

Autobiographical memories are memories of the events from one’s life that make up an individual’s life story (e.g., Tulving, 2001; Fivush, 2011). An essential feature of AMs is their emotionality (Holland and Kensinger, 2010), which at the most basic level is typically described in terms of valence and intensity (e.g., Talarico et al., 2004; Wolf et al., 2021). Valence describes the quality of an emotional experience, that is whether something is positive or negative (or neutral), and is, thus, a categorical variable. Intensity complements the valence dimension by indicating the magnitude or strength of the positive or negative emotional experience and therefore is a continuous and quantitative variable. Notably, emotionality is a time-sensitive feature of AMs (e.g., Hoehne, 2023), meaning that both the intensity and valence associated with events can change over time. In particular, such changes occur as individuals tend to reinterpret a past event in the light of their current situation at the time of recall (Peters et al., 2022). For instance, a relationship break-up may have been perceived as very negative when it happened. After some time, however, one may have come to believe that it was the right thing to do, so that they now perceive the break-up as something positive. This type of change, a change in valence from then to now, is referred to as flexible affect (Walker and Skowronski, 2009; Hoehne and Zimprich, 2024) and can occur in both directions, from positive to negative valence and vice versa. More typically, however, AMs either do not change at all, which is referred to as fixed affect, or they change only in terms of their positive and negative affect intensity, which can take the form of an increase, referred to as flourishing affect, or a decrease, referred to as fading affect (Ritchie et al., 2009; Walker and Skowronski, 2009).

A widely studied, robust phenomenon of emotional change in AMs is the *fading affect bias* (FAB: Walker et al., 1997). Specifically, the FAB names the phenomenon for which, on average, the affect intensity associated with negative AMs fades more strongly and more quickly over time than the affect intensity associated with positive AMs, which sometimes even increases on average. Therefore, by definition, the FAB leads to a rosier perception of the past, and is, thus, considered to reflect a self-serving, emotion-regulating mechanism to support a positive and optimistic view of the self, the world, and the future (Walker and Skowronski, 2009).

Despite the term “*fading* affect bias,” the FAB is not only composed of memories that have faded in affect intensity, but rather is the result of biases in all four types of affect change (for a review, see Skowronski et al., 2014). To illustrate, Hoehne (2023), who separately analysed both the positive and negative affect intensity of the same memories, has recently found that not only was the negative versus positive affect intensity of AMs more likely to fade across AMs, it was also more likely to show flexible affect, in this case from negative to positive valence. Moreover, Hoehne (2023) found that the positive affect intensity of AMs was more likely to show fixed affect or flourishing affect. Notably, flourishing affect was even more common than fading affect for the positive affect intensity of AMs. In another study, Hoehne and Zimprich (2024) replicated and extended these findings by showing that within those AMs that showed fading affect, the magnitude of fading was stronger for negative compared to positive affect intensity, whereas within those AMs that showed flourishing affect, the magnitude of flourishing was greater for positive compared to negative affect intensity.^1^

In summary, therefore, the FAB can be examined in two different ways and at two different levels. Specifically, the change in the intensity of positive and negative AMs can be calculated and averaged, which directly examines the FAB. Alternatively, to more closely examine the composition of the FAB, the different ways that affect changes for each event can be examined, which provides a more granular analysis than a calculated change-score measure. In particular, besides providing a deeper understanding of the composition of the FAB, a major advantage of examining the different types of affect change in AMs separately, rather than examining one continuous FAB score, is that the former acknowledges that different directions of change in AMs may be qualitatively different from each other and, thus, differently related to predictor variables, whereas the latter assumes that, for instance, fading affect would be the exact opposite of flourishing affect and that both could be equally predicted by the same set of variables. However, there are also several advantages to examining a combined FAB score, so rather than arguing about which approach is better, it is more useful to view the two approaches as complementary. The present study examined the different types of affective change separately.

Most commonly, the FAB has been operationalized as a difference score between the AM’s affect intensity at event occurrence and at event recall (for a review, see Skowronski et al., 2014). These difference scores have then been used to obtain the magnitude of the total FAB (e.g., Marsh and Crawford, 2024) or the probability and strength of the different types of affect change (Hoehne, 2023; Hoehne and Zimprich, 2024), as well as the influence of various predictor variables on either the total FAB (e.g., Muir et al., 2022) or the different types of affect change (e.g., Gibbons and Rollins, 2016). With respect to the latter, that is, the investigation of predictor variables of the different types of affect change, the most extensively researched predictor to date is initial affect intensity of the AM (e.g., Gibbons and Rollins, 2016; Hoehne, 2023; Hoehne and Zimprich, 2024).

Initial intensity is of interest in the study of emotional change in AMs because it may relate to the direction and magnitude of change in two possible ways. First, it is possible that high-intensity events change differently from low-intensity events due to cognitive and emotional processes. However, initial intensity is also related to affect change in AMs through the influence of floor and ceiling effects. For instance, a high-intensity positive event cannot show flourishing affect due to scale limitations. This potential confounding effect is well known in the FAB research community, and is most often addressed by statistically controlling for initial intensity in the analyses (Skowronski et al., 2014). However, this control does not separate the potential influence of cognitive and emotional processes from floor and ceiling effects. A possible solution to investigate the association between initial intensity and affect change in AMs without this methodological limitation could be to avoid using scales to measure emotional intensity then and now and to directly ask the participant whether and in what direction a reported AM has changed emotionally (Hoehne and Zimprich, 2024). In the remainder of this manuscript, we will refer to the directly assessed change as *subjective change* and to the quantitative change based on difference scores as *calculated change*.

Subjective affect changes in AMs may complement calculated changes in other respects besides the examination of the effects of initial intensity. To expand on this idea, it may be useful to elaborate on the potential differences between the two measures. In this regard, it may be helpful to consider some of the typical concerns raised in response to the retrospective methods in FAB research. Notably, both types of operationalization of affect change in AMs – subjective change and calculated change – involve retrospective methods. Specifically, for calculated change, participants indicate in the same session how they perceived the emotionality of an event when it occurred and how they would rate it now, and for subjective change, participants are asked directly how their affect associated with an event has changed since it occurred.

Previous studies have shown that the FAB cannot be fully accounted for by retrospective methodology (e.g., Walker et al., 2003; Skowronski et al., 2004; Ritchie et al., 2009). However, it would be equally incorrect to assume that the FAB is unaffected by it. For example, a key concern raised about the retrospective methodology is that participants may overestimate the initial intensity of particularly negative events, leading to an overestimation of the FAB (e.g., Marsh and Crawford, 2024). However, Ritchie et al. (2009) conducted a diary study in which they showed that participants underestimated rather than overestimated the FAB through the use of retrospective methods. This finding suggests that, while retrospective bias cannot account for the FAB, it still affects it to some extent. Particularly, this discrepancy between longitudinal and retrospective ratings of affect change may differentiate subjective and calculated change.

Specifically, AMs are shaped by current feelings, goals, and ideas about the self (Conway and Pleydell-Pearce, 2000), and are therefore subjective in nature. Consequently, what people remember may (a) be different from and (b) determined by different processes than what would have been measured longitudinally. The former, however, may be even more relevant to an individual’s self-concept, as it potentially reflects their personal truth more closely. Unlike the retrospective assessment of calculated change in AMs, the assessment of subjective change in AMs does not require individuals to rate the initial intensity of events retrospectively. Instead, it relies entirely on how individuals think that their feelings have changed. Therefore, even more than calculated change, it may reflect perceived change in AMs, that is, how an individual perceives their past, regardless of how they originally perceived it when it occurred. Examining subjective changes may, therefore, offer a new perspective on the FAB, complementing previous research using difference scores.

Another typical concern with the previous retrospective methodology is that retrospective ratings of change may emerge due to personal theories about how the emotionality of events generally changes over time. Two previous studies have addressed the question of whether personal theories of emotional change in AMs could account for the FAB in calculated change (Ritchie et al., 2009; Crawford and Marsh, 2023), and found no evidence to support this possibility. Specifically, Crawford and Marsh (2023) examined participants’ theories of affect change in AMs in three different studies. Across all three studies, they found that participants expected both negative and positive affect to fade over time, but they consistently expected positive affect to fade more strongly and faster than negative affect, which would be the opposite of what was observed in the FAB. Crawford and Marsh (2023), therefore, concluded that personal theories of emotional change in AMs cannot explain the FAB. However, if subjective change was more strongly determined by an individual’s active monitoring, these ratings might have been more strongly influenced by typical personal theories of emotional change in AMs. Therefore, if subjective change demonstrated a FAB about equivalent to that of calculated change, this outcome would further support the notion that personal theories cannot account for the FAB, and, thus, also the appropriateness of retrospective methods in FAB research. Consequently, subjective changes complement calculated changes in this respect again.

Other cognitive processes or individual factors than retrospective bias or personal theories of emotional change may be involved in how and whether potential differences in subjective versus calculated change emerge. One step towards a better understanding whether and how subjective and calculated change might differ may be to compare their association with some adaptive and non-adaptive individual difference variables. To illustrate, previous studies of calculated change in AMs have found that the magnitude of the FAB (operationalized by difference scores) was increased with higher levels of adaptive psychological traits, such as dispositional mindfulness (Crawford et al., 2023) and grit (Walker et al., 2020), whereas it was attenuated with higher levels of non-adaptive psychological traits, such as anxiety (Walker et al., 2014) or dysphoria (Walker et al., 2003). In terms of the individual types of affect change, the results point in the same direction as the FAB results that calculated changes in affect are generally more positive with higher levels of adaptive psychological traits and more negative or less positive with higher levels of non-adaptive psychological traits (Hoehne, 2023; Hoehne and Zimprich, 2024). It would be interesting to examine whether the same pattern of effects emerges when assessing subjective change rather than calculated difference scores.

Two psychological traits were selected for the present study: neuroticism and life satisfaction. Neuroticism, by definition, reflects a disposition to negative affect (Tellegen, 1985). Some previous studies have statistically controlled for the effects of neuroticism on the FAB, but the results have been mixed (e.g., Gibbons et al., 2015, 2021, 2024). Muir et al. (2022) directly examined the relation of neuroticism to the FAB and found that high levels of neuroticism were associated with less fading of negative AMs and thus a small FAB. Furthermore, neuroticism is associated with, for instance, feelings of anxiety, depression and vulnerability to stress (Costa and McCrae, 1995), and high levels of (pathological) anxiety and dysphoria have been associated with a weak FAB in previous studies (Walker et al., 2003; Walker et al., 2014). Therefore, we expected neuroticism to be associated with an increased likelihood of subjective change in a negative direction and/or, vice versa, a decreased likelihood of subjective change in a positive direction.^2^

In addition, we included life satisfaction, which is the cognitive-evaluative component of subjective wellbeing (Diener, 1984). In Hoehne and Zimprich (2024), life satisfaction was associated with an increased likelihood of reporting AMs that flourished in positive affect and AMs that changed from negative to positive valence compared to reporting AMs that remained fixed. We, therefore, expected that high levels of life satisfaction would be associated with a high likelihood of subjective change in a more positive direction, and/or a low likelihood of subjective change in a more negative direction.

In summary, subjective assessment of emotional change in autobiographical memory may differ from calculated change in AMs, thus opening up a new window on the FAB and its predictors, which have almost exclusively been operationalized using difference scores. Therefore, the goal of the present study was to investigate subjective change in positive and negative AMs from a variety of different perspectives. Specifically, the first goal of the present study was to examine whether the pattern of bias typically found in calculated change (Hoehne, 2023; Hoehne and Zimprich, 2024) also emerges when assessing subjective change in AMs. Second, because the use of difference scores does not allow for the investigation of the unbiased effect of initial intensity on affect change in AMs due to scale limitations, the present study aimed to examine the effects of initial intensity on subjective change in positive and negative AMs, and compared them with the effects on calculated change. Finally, the third goal of the present study was to test how individual levels of neuroticism and life satisfaction relate to the likelihood of each type of subjective affect change in positive and negative AMs, and whether these associations differ from those with calculated change. To this end, the present study analysed a large adult lifespan sample in which participants freely recalled AMs of positive and negative events and retrospectively rated their affect intensity from the perspective of the event and from the present perspective at the time of recall, as well as whether and in what direction these events had subjectively changed. Each participant reported up to six AMs, resulting in hierarchical data where AMs are nested within participants. To account for this nested data structure, multilevel models were used, allowing the influence of predictor variables to be examined at two levels, that is at the event level (Level 1) and at the participant level (Level 2).

## 2 Method

### 2.1 Sample and procedure

The present data were collected online as part of a large project on psychological constructs across the adult lifespan between November 2023 and February 2024. The study was performed in line with the principles of the Declaration of Helsinki and was deemed exempt from full application for ethical approval by the Ethics Committee of Ulm University. Participants were recruited through email, word-of-mouth, and promotional flyers. The final sample of the present study consisted of *N* = 2,163 adults aged 18 to 98 years (*M* = 46.53 years, *SD* = 17.90 years)^3^^,^^4^. Slightly overrepresented were women (58.79% women, 0.52% gender diverse, 0.41% who did not wish to disclose their gender), and participants who reported at least 3 years of study (53.52%).

Participants completed the study via SoSci Survey (Leiner, 2024).^5^ First, they were presented with the study information and signed the informed consent form, and were subsequently asked to provide subjective health (physical and mental) and demographic information (age, gender, education, marital status, first language, population of their place of residence, whether they lived alone). They were then administered, in a randomized order, several individual difference questionnaires, including the Temporal Satisfaction with Life Scale (Pavot et al., 1998) as a measure of life satisfaction and the BFI-K (Rammstedt and John, 2005) as a measure of neuroticism,^6^ and an autobiographical memory task. Ultimately, participants indicated whether they had answered all the questions honestly and carefully, and could choose to participate in a draw for an online-voucher (worth 50, 30 or 20 Euros).

### 2.2 Measures

#### 2.2.1 Autobiographical memory task

Prior to the actual autobiographical memory test, participants were instructed that the memories to be recalled should relate to distinct events from their past that were older than 1 year, and did not necessarily have to be extraordinary.^7^ Participants then spontaneously recalled three AMs of events that were overall more positive when they occurred, and three AMs of events that were overall more negative when they occurred. Participants were randomized as to whether they described the three positive or the three negative events first (one at a time). After recalling each event, participants gave a brief description of the event and answered some questions about the event in a randomized order.

In this regard, participants rated whether and in which direction the affect intensity of each event had changed subjectively. Each event was separately rated on *subjective change* in both positive and negative affect intensity. For the present analyses, subjective change of positive affect intensity was used for positive AMs and subjective change of negative affect intensity for negative AMs.^8^ The items for subjective change of positive and negative affect intensity are shown in Table 1. Participants also indicated the *initial positive and negative affect intensity* of each event (e.g., “How positive did you feel about the event at the time it happened?”) and their positive and negative affect intensity from a current perspective (e.g., “How positive would you rate the event emotionally today?”) on a scale ranging from *not at all* (1) to *very much* (7). Again, positive affect intensity ratings were used for positive AMs and negative affect intensity ratings for negative AMs. The order in which participants first rated the subjective change or affect intensity then and now in an AM, was randomized across participants.

**Table 1:** Items used to assess subjective change in positive and negative AMs.

Construct	Item
Positive affect intensity of positive AMs	
Would you say that your positive feelings about this event have changed?	
Flourishing Affect	Yes, today I remember the event even more positively
Fading Affect	Yes, today I remember the event less positively
Fixed Affect	No, I remember the event as positively as when it happened
Flexible Affect	Yes, at the time I experienced the event as rather positive, but now I see it as rather negative
Negative affect intensity of negative AMs	
Would you say that your negative feelings about this event have changed?	
Flourishing Affect	Yes, today I remember the event even more negatively
Fading Affect	Yes, today I remember the event less negatively
Fixed Affect	No, I remember the event as negatively as when it happened
Flexible Affect	Yes, at the time I experienced the event as rather negative, but now I see it as rather positive

To classify each AM into a type of calculated change, a difference score was constructed for each AM between the affect intensity rating from the event perspective and the affect intensity rating from a present perspective. If the positive or negative affect intensity decreased, the AM was categorized as fading affect. Moreover, an AM was categorized as flourishing affect if it increased in affect intensity and as fixed affect if it did not change. For a detailed description of this procedure, see Hoehne and Zimprich (2024). Finally, if an AM had a higher initial positive than negative affect intensity rating, but at recall the negative affect intensity rating was higher than the positive one, it was categorized as flexible affect from positive to negative valence. The same general procedure was used to categorize flexible affect from negative to positive valence.

#### 2.2.2 Neuroticism

The BFI-K (Rammstedt and John, 2005) is an abbreviated version of the Big Five Inventory (John et al., 1991), which, according to previous research, has good psychometric properties and is a reliable and valid instrument for measuring the Big Five personality factors in an economic time frame (Rammstedt and John, 2005; Kovaleva et al., 2013). In the present study, the German version of the scale (Rammstedt and John, 2005) was used to measure the Neuroticism subscale, which consists of four items. Participants were asked to respond to each item on a scale ranging from *strongly disagree* (1) to *strongly agree* (7). All positively worded items were reverse scored. Cronbach’s alpha for the neuroticism subscale was 0.81. On average, participants reported moderate values of neuroticism (*M* = 3.92, *SD* = 1.27).

#### 2.2.3 Life satisfaction

The Temporal Satisfaction with Life Scale (Pavot et al., 1998) was adapted from the Satisfaction with Life Scale (Diener et al., 1985) and measures an individual’s past, present and future life satisfaction. The scale is widely used in research and shows good psychometric properties (McIntosh, 2001). For the present analysis, only present life satisfaction was used. The present life satisfaction subscale consists of 5 items presented in a randomized order. In the present study, the German translation by Schuhmacher (2003) was used. Participants rated their agreement with each item on a scale, ranging from *strongly disagree* (1) to *strongly agree* (5). An example item is: “The current conditions of my life are excellent.” Cronbach’s alpha for present life satisfaction in the present study was 0.92. On average, participants were moderately satisfied with their lives (*M* = 4.73, *SD* = 1.35).

### 2.3 Statistical approach

The goal of the present study was to examine the probabilities of the four categories of subjective change in positive and negative AMs and how they differ from the four categories of calculated change, as well as to examine how both subjective and calculated change are associated with specific event and participant level predictor variables. All analyses were conducted separately for positive and negative AMs and for subjective change and calculated change.

Mixed effects multinomial models (Hedeker, 2003; Hedeker and Gibbons, 2006) were used to analyze the probabilities of the four affect change categories, which allows the probability of the unordered categories of a dependent variable to be modeled.^9^ To estimate a multinomial regression model, a reference category must be determined. Fixed affect, which is no change, was chosen as the reference category, so that the probability of any type of change is compared to no change (see also Hoehne, 2023; Hoehne and Zimprich, 2024). More specifically, in our particular case, multinomial regression can be thought of as the simultaneous estimation of logistic regression models for the comparisons between (1) fading versus fixed affect, (2) flourishing versus fixed affect, and (3) flexible versus fixed affect---but with increased statistical power compared to running three logistic regression analyses (Stroup, 2012). In addition, the multinomial regression model was extended to include random intercept effects, as the structure of the present data was multilevel in nature (AMs nested within participants) (see Hedeker, 2003; Hedeker and Gibbons, 2006). In particular, individuals typically differ in the probabilities with which they report AMs with fixed, flourishing, fading, and flexible affect change, which is accounted for by including random intercepts. A more detailed description of the mixed-effects multinomial regression model is provided by Zimprich and Wolf (2018).

Due to the multilevel structure, control and predictor variables could enter the models at two different levels. Participant characteristics, that is age, gender (0 = male, 1 = female, 3 = other),^10^ education (whether participants had at least 3 years of study), neuroticism, and life satisfaction, entered the models at the participant level (Level 2) and were centered across participants (grand-mean centering). The memory level predictor, initial intensity, and control variable, time since event, entered the models at the memory level (Level 1) and were centered within participants (group-mean centering).

All analyses were conducted using SAS NLMIXED (SAS Institute Inc., 2014). Akaike’s Information Criterion (AIC; Akaike, 1974) was used as a measure of model fit, which is based on minus two times the log-likelihood (−2LL) of the data added by a penalty factor for additional parameters, thus rewarding parsimony (see also Vrieze, 2012). The alpha-level for statistical significance was set at 0.05.

## 3 Results

### 3.1 Descriptive statistics

A total of 12,314 AMs (6,157 positive and 6,157 negative) were used for the analyses. Positive AMs were on average rated as high in initial affect intensity (*M* = 6.44, *SD* = 0.97), as were negative AMs, albeit with lower total values (*M* = 6.25, *SD* = 1.18) (*t* (2161) = 9.63, *p* < 0.001). The absolute and relative frequencies of each type of subjective change and calculated change in positive and negative AMs are shown in Table 2.

**Table 2:** Frequency of each type of subjective affect change and calculated change in positive and negative AMs.

Type of change	Positive AMs				Negative AMs			
	Subjective change		Calculated change		Subjective change		Calculated change	
	*n*	%	*n*	%	*n*	%	*n*	%
Fixed Affect	3,820	62.0	4,080	66.3	2,947	47.9	2,651	43.1
Flourishing Affect	1,575	25.6	1,029	16.7	837	13.6	446	7.2
Fading Affect	631	10.3	910	14.8	1878	30.5	2,447	39.7
Flexible Affect	131	2.1	138	2.2	495	8.0	613	10.0
Total	6,157	100.0	6,157	100.0	6,157	100.0	6,157	100.0

*N* = 2,163 individuals, *n* = 12,314 AMs.

For subjective affect change, fixed affect was the most common type of change within both positive and negative AMs, but it was even more common in positive AMs. Similarly, subjective flourishing affect was about twice as common in positive AMs as in negative AMs. Subjective fading affect was about three times more common in negative AMs than in positive AMs, and for flexible affect, which was the least common type of subjective change in both positive and negative AMs, a change from negative to positive valence was about four times more common than a change from positive to negative valence.

For calculated change, the same general pattern emerged. However, compared to calculated change, subjective change was more likely to show flourishing affect and less likely to show fading affect for both positive and negative AMs. Specifically, positive AMs were approximately 9% more likely to show flourishing affect for subjective change compared to calculated change, whereas they were approximately 4% less likely to show fading affect. Similarly, flourishing of negative AMs was approximately 6% more likely and fading of negative AMs was approximately 9% less likely for subjective change compared to calculated change. In addition, for positive AMs, fixed affect was about 4% less likely for subjective change compared to calculated change, whereas for negative AMs, fixed affect was about 5% more likely. Finally, flexible affect from positive to negative valence was about equally likely for subjective and calculated change, but flexible affect from negative to positive valence was slightly (~ 2%) more likely for calculated change than for subjective change. An analysis of these marginal probabilities using a log-linear model for repeated multinomial variables showed that the distributions of subjective and calculated change were significantly different (positive AMs: χ^2^ = 458.63, *df* = 3, *p* < 0.0001; negative AMs: χ^2^ = 328.37, *df* = 3, p < 0.0001). For both positive and negative AMs, the differences between subjective and calculated change amounted to large effects, which were mainly driven by the differences in flourishing affect in positive AMs and both flourishing and fading affect in negative AMs.

### 3.2 Multilevel multinomial model

First, four baseline models (Model 0) were estimated, one for subjective change in positive AMs, one for calculated change in positive AMs, one for subjective change in negative AMs, and one for calculated change in negative AMs. The models included only fixed and random intercepts. The parameter estimates from the two positive AM models are shown in Table 3, and parameter estimates from the two negative AM models are shown in Table 4.

**Table 3:** Results of mixed effects multinomial model 0 for subjective change and calculated change of positive AMs.

Parameter	Flourishing		Fading		Flexible	
	Subjective change	Calculated change	Subjective change	Calculated change	Subjective change	Calculated change
Intercept	−1.138*	−1.733*	−2.280*	−1.787*	−4.483*	−4.559*
Random Effects						
Intercept Variance	2.013*	1.626*	2.365*	1.373*	3.551*	3.636*
R^2^	0.000	0.000	0.000	0.000	0.000	0.000
Intercept correlations subjective change:			Intercept correlations calculated change:			
Flourishing with Fading		0.614*	0.497*			
Flourishing with Flexible		0.385*	0.427*			
Fading with Flexible		0.695*	0.717*			
Model fit subjective change			Model fit calculated change			
-2LL		11,369	11,269			
AIC		11,387	11,287			

Reference category = fixed affect; *N* = 2,163 individuals, *n* = 6,157 AMs. *p < 0.05 (two-tailed); All within-person variables have been centered around the group-mean, all between-person variables have been centered around the grand-mean; Intraclass Correlation Subjective Change: Flourishing = 0.38, Fading = 0.42, Flexible = 0.52; Intraclass Correlation Calculated Change: Flourishing = 0.33, Fading = 29, Flexible = 0.52.

**Table 4:** Results of mixed effects multinomial model 0 for subjective change and calculated change of negative AMs.

Parameter	Flourishing		Fading		Flexible	
	Subjective change	Calculated change	Subjective change	Calculated change	Subjective change	Calculated change
Intercept	−1.478*	−2.163*	−0.486*	−0.044	−2.618*	−1.810*
Random Effects						
Intercept Variance	3.015*	1.844*	2.378*	1.614*	4.192*	2.851*
R^2^	0.000	0.000	0.000	0.000	0.000	0.000
Intercept correlations subjective change:			Intercept correlations calculated change:			
Flourishing with Fading		0.808*	0.434*			
Flourishing with Flexible		0.627*	0.501*			
Fading with Flexible		0.539*	0.797*			
Model fit subjective change			Model fit calculated change			
-2LL		14,007	13,788			
AIC		14,025	13,806			

Reference category = fixed affect; *N* = 2,163 individuals, *n* = 6,157 AMs. *p < 0.05 (two-tailed); All within-person variables have been centered around the group-mean, all between-person variables have been centered around the grand-mean; Intraclass Correlation Subjective Change: Flourishing = 0.48, Fading = 0.42, Flexible = 0.56; Intraclass Correlation Calculated Change: Flourishing = 0.36, Fading = 33, Flexible = 0.46.

For *subjective change* in *positive* AMs, the intercept estimate for flourishing affect was *β*_0*FlourishP*_ = −1.138. Translated back to the probability scale, the conditional probability for flourishing affect was


$$\begin{array}{l} p_{\text{FlourishP}}=\frac{\exp(\beta_{0\text{FlourishP}})}{1+\exp(\beta_{0\text{FlourishP}})+\exp(\beta_{0\text{FadeP}})+\exp(\beta_{0\text{FlexibleP}})}\\ =\frac{\exp(-1.138)}{1+\exp(-1.138)+\exp(-2.280)+\exp(-4.483)}=.223 \end{array}$$

or 22.3%. Similarly, the conditional probability for fading affect was 7.1%, for flexible affect 0.8%, and, consequently, for fixed affect 69.7%. Notably, these probabilities differ slightly from the observed relative frequencies (Table 2) due to differences between marginal and conditional effects. Specifically, the observed (marginal) distribution of the outcome variable is different from the distribution of the outcome variable conditional on the random effects (Stroup, 2012), and with increasing random variance, the difference increases between these two distributions. All random intercept variances were significant, indicating that participants differed reliably in their probability of reporting AMs from the different types of subjective affect change. Intraclass correlations, that is, the total variance in each individual comparison with fixed affect reflecting differences between participants, are shown in Table 3. Specifically, between 38.0% (fading affect) and 51.9% (flexible affect) of the total variance was due to between-person differences in the subjective change of positive AMs, which can be interpreted as a large amount of between-person variance. In addition, all random intercepts were significantly correlated with medium (flourishing with flexible affect) to strong effects (flourishing with fading affect and fading with flexible affect), indicating that participants tended to report either subjective change or no subjective change in positive AMs in general.

The results for *calculated change* in *positive* AMs were comparable to those for subjective change. All random intercept variances and random intercept correlations were significant (see Table 3). However, the conditional probabilities were somewhat different from the subjective change model. Specifically, the conditional probabilities for calculated change were 13.0% for flourishing affect, 12.4% for fading affect, 0.8% for flexible affect, and 73.8% for fixed affect. Intraclass correlations ranged from 29.4% (fading affect) to 52.5% (flexible affect) and were lower in flourishing and fading affect for calculated change than subjective change, indicating that the variance in calculated change was less driven by differences between participants than the variance in subjective change.

In the *subjective change* model of *negative* AMs (Table 4), the conditional probabilities were 11.9% for flourishing affect, 32.1% for fading affect, 3.8% for flexible affect, and 52.2% for fixed affect. All random intercept variances and intercept correlations were again significant. Intraclass correlations indicated that between 42.0% (fading affect) and 56.0% (flexible affect) of the total variance in the comparisons with fixed affect were due to between-person differences in subjective change of negative AMs.

Again, the results for *calculated change* in *negative* AMs were relatively comparable to those for subjective change in negative AMs, except for the conditional probabilities, specifically, 5.1% for flourishing affect, 42.8% for fading affect, 7.3% for flexible affect, and 44.7% for fixed affect. All random intercept variances and random intercept correlations were significant as well. Intraclass correlations ranged from 32.9% (fading affect) to 46.4% (flexible affect), and, as with positive AMs, were lower than those for subjective change.

In Model 1, control and predictor variables were added. The parameter estimates for the positive AM models of subjective change and calculated change are shown in Table 5, and the parameter estimates for the negative AM models of subjective change and calculated change are shown in Table 6. In the subjective change model of positive AMs, as indicated by R^2^, the control and predictor variables together accounted for between 5.8% (fading affect) and 17.4% (flourishing affect) of the total variance in the comparison with fixed affect, whereas between 5.6% (fading affect) and 29.4% (flourishing affect) of the total variance were accounted for in the calculated change model of positive AMs. Furthermore, in the subjective change model of negative AMs, the control and predictor variables together accounted for between 4.3% (fading affect) and 16.3% (flourishing affect) of the total variance in the comparisons with fixed affect, whereas between 6.9% (fading affect) and 37.5% (flourishing affect) of the total variance were accounted for in the calculated change model of negative AMs. Below, the results are presented separately for each predictor variable.

**Table 5:** Results of mixed effects multinomial Model 1 for subjective change and calculated change of positive AMs.

Parameter	Flourishing		Fading		Flexible	
	Subjective change	Calculated change	Subjective change	Calculated change	Subjective change	Calculated change
Intercept	−1.285*	−2.801*	−2.290*	−1.795*	−4.405*	−4.574*
Within person variables						
Time since event	0.004*	0.013†	0.011*	0.012*	0.014*	0.019
Initial intensity	−1.030*	−3.097*	−0.266*	0.022	−0.374*	−1.014*
Between person variables						
Age^○^	−0.019*	−0.036*	−0.021*	−0.018*	−0.019*	−0.026*
Gender	0.186	0.154	−0.110*	−0.307*	0.485*	0.211
Education	0.051*	0.356*	0.319*	0.336*	−0.502*	−0.210
Neuroticism	0.022^†^	0.000	0.041*	0.032*	−0.021*	0.041
Life Satisfaction	0.001*	−0.015	−0.048*	−0.038*	−0.062*	−0.053*
Random effects						
Intercept Variance	2.733*	6.153*	2.300*	1.158*	3.162*	4.017*
*R* ^2^	0.174	0.294	0.058	0.056	0.098	0.194
Intercept correlations subjective change:			Intercept correlations calculated change:			
Flourishing with Fading		0.610*	0.248*			
Flourishing with Flexible		0.391*	0.488*			
Fading with Flexible		0.764*	0.674*			
Model fit subjective change			Model fit calculated change			
-2LL		10,815	9,525			
AIC		10,875	9,585			

Reference category = fixed affect; *N* = 2,163 individuals, *n* = 6,157 AMs. *p < 0.05 (two-tailed), ^†^*p* < 0.10 (two-tailed); All within-person variables have been centered around the group-mean, all between-person variables have been centered around the grand-mean; ^○^Variable has been divided by 10 to achieve numerically larger and more stable estimates; *R*^2^ calculated according to Jaeger et al. (2017).

**Table 6:** Results of mixed effects multinomial Model 1 for subjective change and calculated change of negative AMs.

Parameter	Flourishing		Fading		Flexible	
	Subjective change	Calculated change	Subjective change	Calculated change	Subjective change	Calculated change
Intercept	−1.617*	−3.705*	−0.494*	−0.049	−2.640*	−1.877^*^
Within person variables						
Time since Event	0.012**	0.033*	0.010**	0.017*	0.010*	0.225*
Initial Intensity	−0.587*	−1.992*	−0.156*	−0.001	−0.375*	−0.583*
Between person variables						
Age^○^	−0.028*	−0.042*	−0.021*	−0.024*	−0.019*	−0.033*
Gender	0.196	0.208	0.110*	0.053	0.238	0.357*
Education	0.281**	0.415*	0.248*	0.338*	0.093	0.263*
Neuroticism	0.012	−0.007	−0.017	−0.012	−0.044**	−0.048*
Life Satisfaction	−0.008*	−0.029†	0.018*	0.026*	0.016	0.036*
Random effects						
Intercept Variance	3.057*	4.197*	2.190*	1.420*	4.152*	2.717*
R^2^	0.163	0.375	0.043	0.069	0.058	0.117
Intercept correlations subjective change:			Intercept correlations calculated change:			
Flourishing with Fading		0.784*	0.269*			
Flourishing with Flexible		0.645*	0.465*			
Fading with Flexible		0.516*	0.747*			
Model fit subjective change			Model fit calculated change			
-2LL		13,705	12,654			
AIC		13,765	12,714			

Reference category = fixed affect; *N* = 2,163 individuals, *n* = 6,157 AMs. *p < 0.05 (two-tailed), †*p* < 0.10 (two-tailed); All within-person variables have been centered around the group-mean, all between-person variables have been centered around the grand-mean; ^○^Variable has been divided by 10 to achieve numerically larger and more stable estimates; *R*^2^ calculated according to Jaeger et al. (2017).

#### 3.2.1 Initial intensity

For subjective change of positive AMs, higher levels of positive initial intensity were associated with a lower probability of an AM exhibiting flourishing affect, fading affect, and flexible affect compared to remaining fixed in affect. Note that a useful calculation to interpret and compare the estimates is transforming them into an odds ratio (OR) for a standardized predictor variable change (Long, 1997). For the effect of initial intensity (centered within-person) on flourishing affect in positive AMs one gets.

$\begin{array}{l} \text{OR}=\exp(\beta_{\text{InitialIntensityFlourishP}}\;x\;SD_{\text{InitialIntensityP}})\\ =\exp(-1.030\;x\;0.699)=0.49 \end{array}$ , indicating that for a one standard deviation change in initial intensity, the odds for this AM to show flourishing compared fixed affect in positive AMs change by a factor of 0.49. Similarly, the ORs for the effects of initial intensity on fading and flexible affect in positive AMs were 0.83 and 0.77, respectively.

For calculated change of positive AMs, higher levels of positive initial intensity were also associated with a lower probability of flourishing affect (OR: 0.11) and flexible affect (OR: 0.49), but with somewhat stronger effect sizes compared to subjective change. In contrast to subjective change, positive initial intensity had no effect on the likelihood of fading affect in calculated change.

Exactly the same pattern emerged for negative AMs. That is, for both subjective change and calculated change, higher levels of negative initial intensity were associated with a lower likelihood of flourishing affect (OR(subjective): 0.60; OR(calculated): 0.18) and flexible affect (OR(subjective): 0.72; OR(calculated): 0.60). Although the effect sizes were substantially larger for calculated change than for subjective change. Again, only for subjective change of negative AMs did initial intensity have an effect on the likelihood of fading affect (OR: 0.87).

#### 3.2.2 Neuroticism

For subjective change of positive AMs, high individual levels of neuroticism were associated with an increased probability of reporting positive AMs that faded in affect intensity compared to reporting positive AMs that remained fixed in affect (OR: 1.23). The same effect was also evident for calculated change of positive AMs (OR: 1.18). Both effects were relatively comparable in size. Otherwise, neuroticism had no effect on either subjective change or calculated change of positive AMs.

For negative AMs, the same pattern with comparable effect sizes emerged for subjective change and calculated change. Specifically, for both measures, high individual levels of neuroticism were associated with a decreased probability of reporting flexible affect AMs that changed from negative to positive valence compared to reporting negative AMs that remained fixed (OR(subjective): 0.80; OR(calculated): 0.78).

#### 3.2.3 Life satisfaction

For positive AMs, the effects for life satisfaction showed the same pattern across subjective change and calculated change with relatively comparable effect sizes. Specifically, higher individual levels of life satisfaction were associated with a decreased probability of reporting both fading affect (OR(subjective): 0.72; OR(calculated): 0.77), and flexible affect from positive to negative valence (OR(subjective): 0.66; OR(calculated): 0.70).

For negative AMs, the effects of life satisfaction differed between subjective change and calculated change. Although high individual levels of life satisfaction were associated with an increased probability of reporting fading affect compared to fixed affect for both measures (OR(subjective): 1.13; OR(calculated): 1.19) with relatively comparable effect sizes, only for calculated change did high levels of life satisfaction significantly and positively affect the probability of reporting flexible affect AMs that changed from negative to positive valence (OR: 1.28).

**Figure 1:**
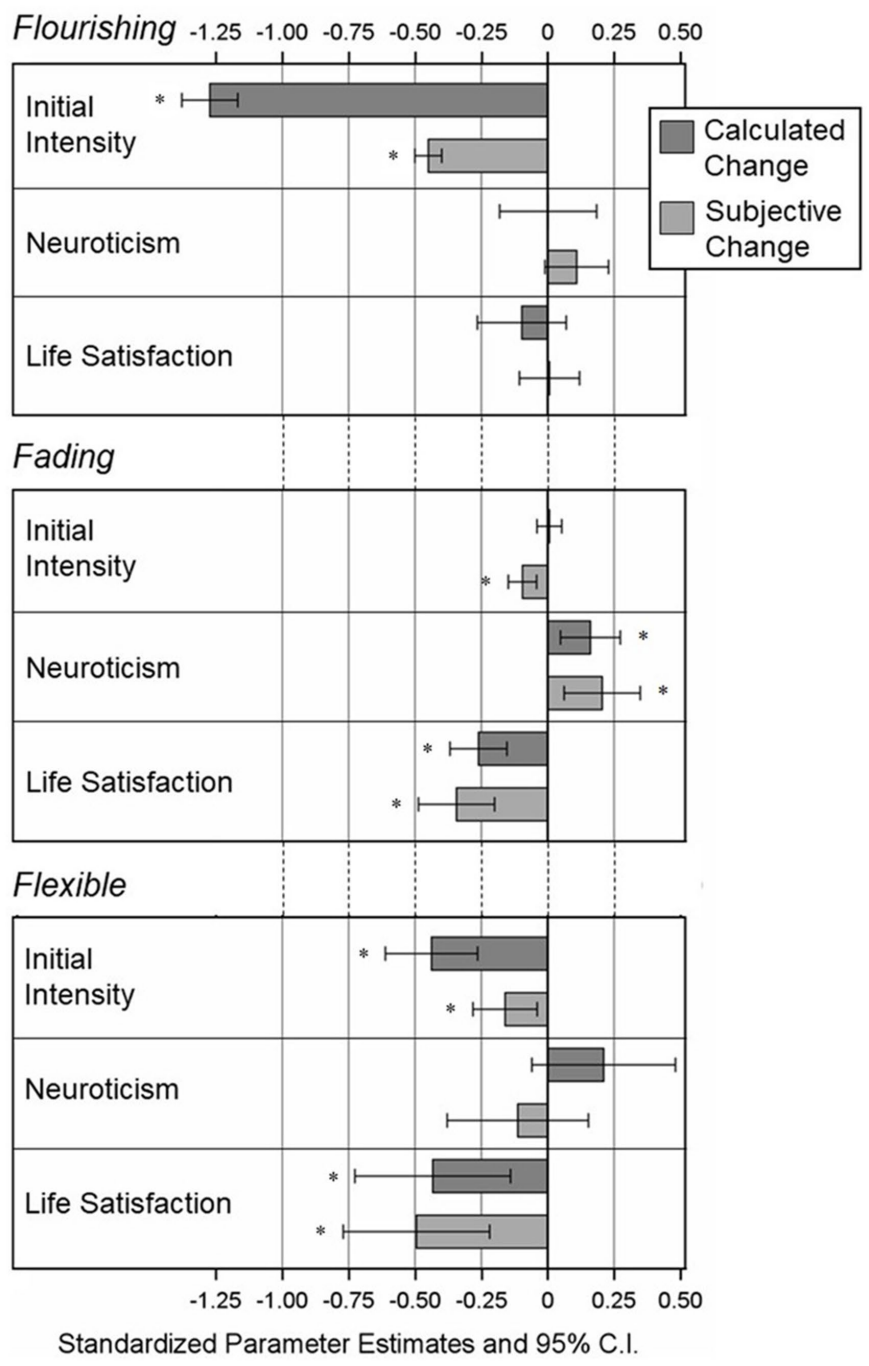
Standardized effects of predictor variables on Subjective change and calculated change in *Positive* AMs from Model 1; Reference Category = Fixed Affect; *N* = 2,163 Individuals, *n* = 6,157 AMs. *p < 0.05 (two-tailed).

**Figure 2:**
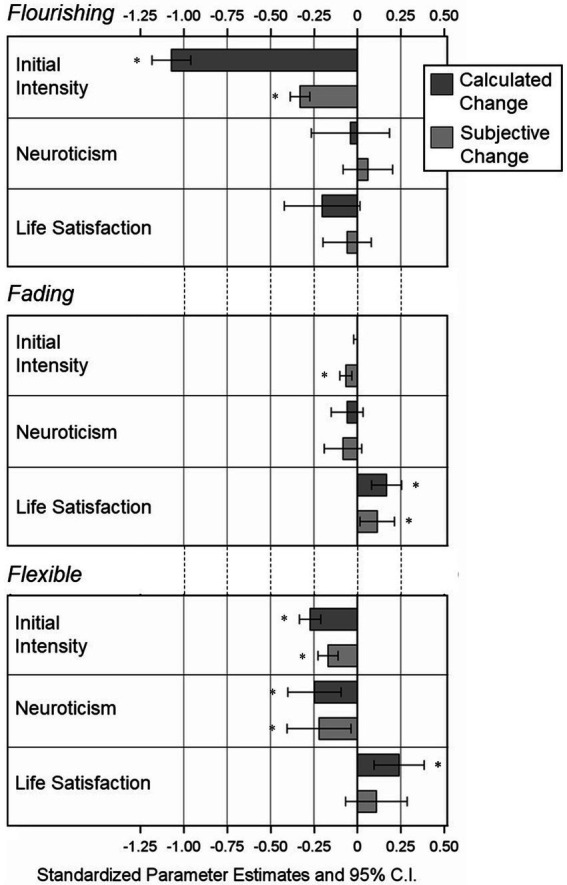
Standardized effects of predictor variables on subjective change and calculated change in *Negative* AMs from Model 1; Reference Category = Fixed Affect; *N* = 2,163 Individuals, *n* = 6,157 AMs. *p < 0.05 (two-tailed).

For a better comparison of the effects and effect sizes, the standardized parameter estimates with 95% confidence intervals for all predictor variables are shown in Figure 1 (positive AM models) and Figure 2 (negative AM models). As can be seen in the figures, for both positive and negative AMs, the effect of initial intensity on flourishing affect in calculated change was by far the largest effect and the most different from the same effect on subjective change, although both effects were statistically significant.

## 4 Discussion

The goal of the present study was to test whether the FAB also appears for subjective change in AMs and whether it differs from the FAB in calculated change. In addition, the present study examined the effects of the predictor variables, initial intensity, neuroticism, and life satisfaction, on the different types of subjective change in AMs and compared them with the effects on calculated change. Below, we discuss the results with regard to the emergence of a FAB, followed by a discussion of the effects of predictor variables.

### 4.1 Evidence of a FAB in subjective change in AMs

In the present data, biases for all four, flourishing affect, fading affect, flexible affect and fixed affect, emerged for both subjective change and calculated change. Specifically, regardless of the type of change examined, flourishing affect and fixed affect occurred more often in positive AMs than in negative AMs, whereas fading affect occurred more often in negative AMs than in positive AMs, and flexible affect occurred more often from negative to positive valence than vice versa. Furthermore, in both cases, these separate biases together formed a FAB. This pattern is consistent with and complements previous work on the four types of calculated affect change in AMs (e.g., Gibbons and Rollins, 2016; Hoehne, 2023; Hoehne and Zimprich, 2024) by showing that it also emerges for subjective change.

Despite both types of change demonstrating a FAB, the relative frequencies of the different types of affect change differed to some extent between subjective change and calculated change, which was particularly evident for flourishing affect and fading affect. Specifically, for both positive and negative AMs, flourishing affect was considerably more common and fading affect was less common for subjective change than for calculated change. The former effect may be explained by the fact that high-intensity AMs were more probable to flourish for subjective change as there were no scale limitations. The AMs reported in the present study were, on average, rated as very high in initial intensity and, therefore, may not have been able to demonstrate flourishing affect when calculated change was examined. With regard to fading affect, which was more common for calculated than subjective change, one possible explanation may be that the threshold for labeling an AM as having faded in affect intensity differed between ratings of subjective change and calculated change. Specifically, for an AM to be placed in the fading affect category for calculated change, it was sufficient for the affect intensity rating from the current perspective to be one point below the rating from the initial perspective on a scale from 1 to 7. It is possible that a more significant decrease in affect intensity was required for an AM to be classified as having subjectively faded in positive or negative affect intensity. Consequently, the fading affect finding may also be interpreted as evidence that the processes behind calculated change and subjective change differ to some extent.

As argued in the introduction to this article, one factor behind the differences between subjective and calculated change may be the influence of personal theories of emotional change in AMs on subjective change. Specifically, it was hypothesized that if personal theories influenced subjective ratings of emotional change in AMs, the pattern of subjective change should be more consistent with the typical pattern of theorized emotional change in AMs, namely that both positive and negative affect intensity fade, but that positive affect intensity fades more and faster than negative affect intensity (Ritchie et al., 2009; Crawford and Marsh, 2023). In the present data, fading affect was less common for subjective than for calculated change, and the FAB was evident for both calculated and subjective change, which suggests that personal theories of emotional change cannot account for the FAB.

In summary, subjective change in AMs clearly showed a FAB, supporting the idea of a self-serving, emotion-regulating functionality of autobiographical memory to support a positive and optimistic view of the self, the world, and the future (Walker and Skowronski, 2009) also within directly assessed emotional changes.

### 4.2 Predictors of subjective change in AMs

The second goal of the present study was to examine the effects of predictor variables on the likelihood of the different types of affect change in subjective change, and to compare these effects with those on calculated change. One promising predictor in this regard is the initial affect intensity of AMs. Specifically, initial intensity is the most widely studied predictor of the different types of affect change (Gibbons and Rollins, 2016; Hoehne, 2023; Hoehne and Zimprich, 2024), but has been examined almost exclusively in the context of calculated change, where its effects have possibly been confounded with scale effects (but see Hoehne (2023) and Hoehne and Zimprich (2024), where flexible affect was examined subjectively). The goal of the present study was, therefore, to examine the unbiased effects of initial intensity on emotional change in AMs, that is, the association without the influence of scale limitations.

#### 4.2.1 Initial intensity

In the present data, for both positive and negative AMs, and for both subjective change and calculated change, high initial intensity values were associated with a decreased probability of an AM showing flourishing affect and flexible affect. For calculated change, the results are fully consistent with the presence of scale limitation effects, and also replicate findings on initial intensity from other studies examining calculated change (e.g., Gibbons and Rollins, 2016). Intriguingly, the effects for subjective change, that is, the effects of initial intensity on emotional change in AMs free from scale limitations, showed the same general pattern in terms of flourishing and flexible affect, albeit with substantially weaker effects, particularly for flourishing affect. We interpret these findings as evidence for a subjective ceiling effect of how good or bad things can get. Specifically, it seems that while some highly intense events may still be subjectively perceived as flourishing, others do not, because an individual may also have a subjective limit of what constitutes maximum affect intensity. Although the flexible affect effects were also weaker for subjective change than for calculated change, the overall result remained the same: High initial intensity events showed a low likelihood of change in valence.

For both positive and negative AMs, initial intensity was not related to the likelihood of fading affect for calculated change, whereas for subjective change, high initial intensity ratings were associated with a low likelihood of fading affect. Previous studies examining calculated change, however, found that high levels of initial intensity ratings were associated with a high probability of fading affect for both positive and negative AMs (Gibbons and Rollins, 2016; Hoehne, 2023; Hoehne and Zimprich, 2024). Specifically, in these previous studies, events that were rated with the lowest affect intensity were unable to fade further due to scale limitations. In the present study, however, all high-intensity events were collected and this restriction of range may explain why initial intensity was not related to fading affect for calculated change. Intriguingly, however, high initial intensity was associated with a decreased probability of fading affect for subjective change. These results suggest that the subjective change measure is more sensitive than the calculated change measure to emotional changes in AMs in the face of extreme initial event affect ratings.

In summary, the present findings suggest that the results are, indeed, different when the effects of initial intensity on emotional change in AMs are examined without the influence of scale effects than when difference scores are used. However, the present findings also point to the existence of a subjective ceiling effect in the emotional intensity of AMs. Future studies may aim to replicate and extend these findings to deepen our understanding of the effects of initial intensity on emotional change in AMs.

#### 4.2.2 Individual characteristics: neuroticism and life satisfaction

As argued in the introduction to this article, individual difference variables may be useful in better understanding potential differences between subjective and calculated changes in AMs.

To this end, we examined the predictive value of two concrete individual characteristics, neuroticism and life satisfaction. On the basis of previous literature, we expected high individual levels of neuroticism to be associated with an increased probability of emotional changes in a negative direction and/or, a decreased probability of emotional changes in a positive direction (Walker et al., 2003; Walker et al., 2014; Muir et al., 2022; Hoehne and Zimprich, 2024). Similarly, on the basis of previous research, we expected high levels of life satisfaction to be associated with an increased probability of emotional changes in a positive direction, and/or a decreased probability of emotional changes in a negative direction (Hoehne and Zimprich, 2024).

In the present data, the effects of neuroticism and life satisfaction were in the expected directions for both subjective change and calculated change. Moreover, despite one effect being only evident for calculated change, the pattern and magnitude of effects were highly comparable between subjective and calculated change (see Figures 1, 2), supporting the idea that both subjective and calculated change may be related to adaptive and non-adaptive individual characteristics in the same or very similar way. However, apart from the effects of neuroticism and life satisfaction, the intraclass correlations, which in this case represent the amount of variance in each single comparison with fixed affect that can be attributed to variance between participants, were systematically higher for subjective change compared to calculated change, suggesting that variations in subjective change are more driven by differences between participants. It is possible that these differences in the prediction of subjective change and calculated change are based on individual difference variables other than neuroticism and life satisfaction. For instance, a closer look at the results for the control variables shows that the effects of gender and education differed more between subjective change and calculated change than the effects of neuroticism and life satisfaction. Future studies could examine the influence of other individual difference variables in predicting subjective change and calculated change in AMs, to improve our understanding of how subjective and calculated change in AMs differ, and what factors might determine these differences.

### 4.3 Limitations and future directions

The present findings suggest that subjective and calculated change in AMs differ to some extent, and therefore complement each other in providing a thorough understanding of the FAB. The patterns observed for subjective and calculated change in the present data did not fit typical personal theories of emotional change in AMs (Crawford and Marsh, 2023), so the differences could not be explained by such personal theories. Moreover, subjective and calculated change were not differentially related to the individual characteristics of neuroticism and life satisfaction. In contrast, the present findings do support the idea that some differences between subjective and calculated change are due to scale limitations, as they are only apparent for calculated change. There may be other mechanisms or cognitive processes not discussed in the present article that could have led to differences between subjective and calculated change, which could be the subject of future research studies. In this respect, it may be particularly fruitful to examine the effects of time since event more closely. In the present data, time since event was quite consistently more strongly related to calculated than subjective change, suggesting that subjective changes are less dependent on the time elapsed since an event. Future studies could further explore the differences between subjective and calculated change by focusing particularly on their association with time since event.

A limitation of the present study is the method of memory sampling used. Specifically, the free recall design almost exclusively sampled high-intensity AMs. As a result, initial intensity had no effect on fading affect in calculated change. However, in previous studies that also sampled low-intensity AMs, initial intensity typically showed a positive association with calculated fading affect. Specifically, low initial intensity was associated with low fading affect (Gibbons and Rollins, 2016; Hoehne, 2023; Hoehne and Zimprich, 2024), which might be explained by scale limitations where low-intensity AMs could not fade further. Future studies could attempt to replicate the present findings on initial intensity using a different, more extensive sample of AMs, which could be accomplished by employing the cue-word technique (Crovitz and Schiffman, 1974; Galton, 1879).

Furthermore, because the present study is the first one to systematically examine subjective changes in AMs, only qualitative changes were analysed as a first step, that is, whether an AM faded, flourished, showed flexible affect or fixed affect in general. However, the FAB is defined as a stronger and faster fading of negative compared to positive affect intensity in AMs, which implies that negative AMs should fade more often (qualitative approach), and to a greater extent than positive AMs (quantitative approach). Similarly, positive AMs should flourish more often and to a greater extent than negative AMs. This exact pattern was demonstrated for calculated change in Hoehne and Zimprich (2024). Consequently, a more extensive comparison of the FAB and its predictors in subjective change and calculated change (or change as measured longitudinally) might compare both qualitative and quantitative changes in AMs, that is, the likelihood and the magnitude of each type of change. Such a combined analysis of qualitative and quantitative changes in AMs would additionally have the advantage over typical previous quantitative approaches to the FAB that it would recognize both that the different directions of change may be qualitatively different from each other (i.e., an increase and a decrease in positive affect intensity may not necessarily be opposites), and that there may also be a different amount of change within each type of change. Therefore, as a next step, future studies could ask participants whether and in what direction an AM has changed emotionally and to what extent it has changed. In this way, future studies could additionally calculate a (latent) FAB score (cf., Hoehne and Zimprich, 2025) to directly compare its magnitude and association with specific predictor variables between subjective change and calculated change (or change as measured longitudinally).^11^ Specifically, Hoehne and Zimprich (2025) recently proposed a new way of modeling the FAB, which involves operationalizing it as a latent construct to account for measurement error and the multilevel structure of the data.

Notably, the present study examined events of a wide age range. The time elapsed since an event may relate to the FAB in two ways. Firstly, research has shown that the FAB increases with event age (Gibbons et al., 2011; Gibbons and Rollins, 2021), though it is lower for events that occurred a very long time ago (Hoehne and Zimprich, 2025). Secondly, time since event may potentially affect the extent to which subjective changes may differ from those that would have been measured longitudinally. Future studies may attempt to replicate the present findings using a sample of more recent events. Furthermore, combining this with diary studies would enable all three types of change – longitudinal, calculated and subjective – to be compared.

Furthermore, although the present sample was quite large and covered a wide age range, it consisted mainly of well-educated individuals, whose first language was German. The present findings should therefore be interpreted with this caveat in mind, and future studies could attempt to replicate the present findings in samples with different demographic backgrounds.

Finally, understanding how individuals subjectively perceive their past, and how this differs from change as measured longitudinally, may have practical implications. In particular, the finding that non-adaptive psychological characteristics are related to negative subjective perceptions of emotional changes in AMs could inform clinical memory interventions. Future studies should investigate ways to manipulate subjective perceptions of AMs to make them more positive, because our perception of our personal past influences our perception of our current self (Conway and Pleydell-Pearce, 2000).

## 5 Conclusion

In a large age-diverse sample, the present study extends previous knowledge of the FAB by demonstrating that it occurs not only for calculated change in AMs, but also for directly assessed subjective change. In addition, the present study extends previous knowledge of the effects of initial intensity on emotional change in AMs, which have been confounded by scale limitations in previous studies. Specifically, the present study found that when examined in a sample of mostly high-intensity events, high levels of initial intensity are associated with a decreased probability of fading affect for subjective change (free from scale limitations) but not for calculated change. Furthermore, the present results suggest a subjective ceiling effect in the emotional intensity of AMs. The patterns observed for neither subjective nor calculated change in the present data fit typical personal theories of emotional change in AMs. Moreover, although neuroticism and life satisfaction had effects in the expected directions, these effects did not differ between subjective change and calculated change. Future studies could further explore potential differences between calculated and subjective change. In conclusion, the present study supports the idea of an emotion-regulating, self-serving functionality of autobiographical memory, also within subjective emotional changes free from scale limitations, which sheds a new, optimistic light on the appropriateness of retrospective designs in FAB studies.

## Data Availability

Data and analysis code are available in an Open Science Framework repository at https://doi.org/10.17605/OSF.IO/BPQNH.

## Footnotes

^1^The reader may have noticed that the operationalization and thus the definition of the FAB in Hoehne (2023) and Hoehne and Zimprich (2024) differs slightly from the definition in the present manuscript. Specifically, whereas in the present manuscript the FAB is defined as, for instance, a stronger fading of the affect intensity of negative versus positive AMs, Hoehne and Zimprich (2024) defined the FAB as a stronger fading of the negative versus positive affect intensity within the same memory. The latter definition is based on the idea and finding that positive and negative affect intensity represent distinct dimensions of emotional experience and can therefore be associated with the same event (Diener and Emmons, 1985; Hoehne and Zimprich, 2024). In contrast, the more traditional definition of the FAB neglects the idea of mixed emotions in AMs. Nevertheless, as the scope of the present study was to introduce a new way of measuring emotional change in AMs (i.e., subjectively measured change), we have retained the traditional definition of the FAB as an initial step in order to make the present results more comparable with previous findings.

^2^Change in a negative direction would be fading affect in positive AMs, flexible affect from positive to negative valence, and flourishing affect in negative AMs. Change in a positive direction are flourishing affect in positive AMs, fading affect in negative AMs, and flexible affect from negative to positive valence.

^3^Originally, 4,639 persons started the online study. Of those individuals, 1,061 (23%) did not provide any data but left the study after the introductory pages. From the remaining 3,578 persons, 920 of them (20%) had to be excluded due to a technical error. More specifically, the autobiographical memory part of the study did not allow participants to enter an age-at-event below 18 years of age during the first 13 days of data collection. From the resulting sample of 2,658 persons, 95 individuals (2%) were excluded because they failed to answer the demographic data questions (age, gender) or did not give informed consent. In the next step, those 181 individuals (4%) who did not answer all five catch items correctly and/ or reported to not have answered carefully and honestly were excluded. From the remaining 2,382 persons, only those individuals who provided at least one positive and one negative AM plus the according emotional intensity (at the time of event and now) and subjective intensity change ratings were included, reducing the sample by 219 (5%) participants. Of these 219 persons excluded in this last step, most of them (69%) had missing values for the negative AMs or did not provide all necessary ratings (31%). The final size of the analysis sample was, thus, *N* = 2,163.

^4^Usually, an *a priori* power analysis is performed to determine the required sample size. However, both the data structure and the analysis model defy any simple power analysis. To ensure sufficient statistical power, it was important to have enough observations for even the smallest type of affect change, namely flexible affect from positive to negative valence. In the present data, 131 AMs showed this type of change for subjective change and 138 AMs showed it for calculated change. In previous studies of the different types of affect change, sufficient power was assumed when at least 50 (Hoehne and Zimprich, 2024) or 100 (Hoehne, 2023) AMs were in the smallest type of affect change. This number has been clearly exceeded in the present data.

^5^ www.soscisurvey.de

^6^Questionnaires that were administered but not included in the present study are the Temporal Focus Scale (Shipp et al., 2009), Herth Hope Index (Herth, 1992), Life Orientation Test (Scheier and Carver, 1985), Rosenberg Self-Esteem Scale (Rosenberg, 1965), the Short Form of the Revised Almost Perfect Scale (Rice et al., 2014), the Future Time Perspective Scale (Carstensen and Lang, 1996) and the Brief Resilience Scale (Smith et al., 2008).

^7^We added the instruction that the events did not have to be extraordinary to ensure that participants did not think that any of their spontaneous memories were not important enough to be reported in the study.

^8^According to Diener and Emmons (1985), positive and negative affect intensity of autobiographical memories represent distinctly different dimensions of human emotional experience and can both be associated with the same event. Therefore, investigating the subjective change in negative affect intensity of positive AMs, or vice versa, is a related but different research topic that was beyond the scope of the present study.

^9^Treating the four affect change categories as ordered would imply that the different AM characteristics are also ordered across the categories. To avoid these restrictions through ordering, we chose a multinomial approach, which is able to treat the different categories as nominal (e.g., Long, 1997).

^10^To center gender, participants who identified as gender diverse or did not wish to disclose their gender were given a value of 0 in the centered binary gender variable, so that they would add to the effects of all predictor variables except gender.

^11^The present data included continuous data for calculated change, so a combined analysis of qualitative and quantitative change could have been applied based on the present data for calculated change. However, in the present study, the purpose of examining calculated change was to serve as a comparison for categorical subjective change, and not to better understand calculated change itself, so it was beyond the scope of the present study to examine qualitative and quantitative calculated change (however, a study that did just that is, Hoehne and Zimprich (2024)).
